# Hepatic lipid droplet-associated proteome changes distinguish dietary-induced fatty liver from glucose tolerance in male mice

**DOI:** 10.1152/ajpendo.00013.2024

**Published:** 2024-04-24

**Authors:** Andries Van Woerkom, Dylan J. Harney, Shilpa R. Nagarajan, Mariam F. Hakeem-Sanni, Jinfeng Lin, Matthew Hooke, Tamara Pulpitel, Gregory J. Cooney, Mark Larance, Darren N. Saunders, Amanda E. Brandon, Andrew J. Hoy

**Affiliations:** ^1^Faculty of Medicine and Health, School of Medical Sciences, Charles Perkins Centre, University of Sydney, Sydney, New South Wales, Australia; ^2^Faculty of Science, School of Life and Environmental Sciences, Charles Perkins Centre, The University of Sydney, Sydney, New South Wales, Australia

**Keywords:** fatty liver, glucose tolerance, lipid droplet, mice, proteomics

## Abstract

Fatty liver is characterized by the expansion of lipid droplets (LDs) and is associated with the development of many metabolic diseases. We assessed the morphology of hepatic LDs and performed quantitative proteomics in lean, glucose-tolerant mice compared with high-fat diet (HFD) fed mice that displayed hepatic steatosis and glucose intolerance as well as high-starch diet (HStD) fed mice who exhibited similar levels of hepatic steatosis but remained glucose tolerant. Both HFD- and HStD-fed mice had more and larger LDs than Chow-fed animals. We observed striking differences in liver LD proteomes of HFD- and HStD-fed mice compared with Chow-fed mice, with fewer differences between HFD and HStD. Taking advantage of our diet strategy, we identified a fatty liver LD proteome consisting of proteins common in HFD- and HStD-fed mice, as well as a proteome associated with glucose tolerance that included proteins shared in Chow and HStD but not HFD-fed mice. Notably, glucose intolerance was associated with changes in the ratio of adipose triglyceride lipase to perilipin 5 in the LD proteome, suggesting dysregulation of neutral lipid homeostasis in glucose-intolerant fatty liver. We conclude that our novel dietary approach uncouples ectopic lipid burden from insulin resistance-associated changes in the hepatic lipid droplet proteome.

**NEW & NOTEWORTHY** This study identified a fatty liver lipid droplet proteome and one associated with glucose tolerance. Notably, glucose intolerance was linked with changes in the ratio of adipose triglyceride lipase to perilipin 5 that is indicative of dysregulation of neutral lipid homeostasis.

## Graphical abstract

**Figure:**
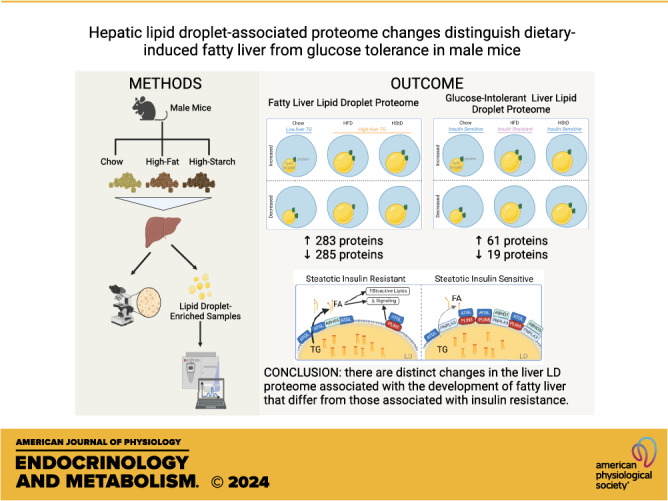

## INTRODUCTION

Fatty liver is an early and defining feature of a range of liver diseases, including non‐alcoholic fatty liver disease [NAFLD (1)], alcoholic liver disease (2), hepatitis C (3), and HIV (4). If not addressed, fatty liver can progress to steatohepatitis, cirrhosis, and carcinoma; in fact, enhanced synthesis of lipids, including fatty acids, glycerolipids, and sphingolipids, is essential for mTORC2-mediated hepatocellular carcinoma (5). It has been estimated that approximately 25% of the world’s population is currently thought to have NAFLD, and this is predicted to increase significantly, leading to increased death due to liver-related pathologies (6). The increasing prevalence of fatty liver reflects the increasing prevalence of other noncommunicable cardiometabolic diseases, such as type 2 diabetes, obesity, and cardiovascular disease (7).

Fatty liver is highly associated with obesity (8); however, the clinical manifestations of obesity are heterogeneous and complex, as is the prevalence of associated disease (9–12). For example, it has been estimated that one-third of patients with obesity are metabolically healthy; the remaining being ‘obese-metabolically unhealthy’ (13), highlighting metabolic diversity within a population defined as obese by BMI. Similarly, it has been suggested that approximately 5–8% of patients with NAFLD in Western countries are considered lean, whereas approximately 20% of the Asian population has lean NAFLD (6). Although currently there are no universally accepted criteria for identifying metabolically-(un)healthy individuals, generally, it includes a combination of adiposity, insulin sensitivity, inflammation, and circulating glucose and lipids (14, 15). Importantly, the incidence of NAFLD is strongly associated with being overweight and obesity, even in well-defined metabolically healthy men and women (16).

NAFLD is defined as intrahepatic triacylglycerol (TG) content greater than 5%, which histologically equates to an increase in the number and size of intracellular lipid droplets (1). Lipid droplets are dynamic organelles that store neutral lipids like triacylglycerol and cholesteryl esters and are coated by a large number of proteins, some of which are known to be involved in the incorporation or release of lipids from the droplets (17). The accumulation of TGs results from an imbalance between the uptake of extracellular lipids, de novo synthesis of fatty acids, oxidation, and release of TG-VLDL (18). In rodent models, lipid accumulation in the liver is an early event in the development of high-fat diet-induced insulin resistance and obesity (19, 20). However, even in animal models, little knowledge exists regarding how lipid storage in liver cells is dysregulated in insulin resistance. Moreover, there is little understanding of the relationship between liver lipid storage and insulin action in a setting of metabolically healthy obesity.

To date, the lipid droplet proteome has been defined for a diverse array of organisms and cell and tissue types [see review Zhang and Liu (17)], including in rodent liver and in models of obesity and NAFLD (21–26). It is important to highlight that these studies predominantly compare two groups, for example, between Chow (control, low-fat diet) and high-fat diet-fed animals or fasting and fasting-refed states, which result in multiple differences between two groups, such as adiposity, tissue and circulating lipid levels, circulating hormone levels, and immune status. This binary normal versus obese framework fails to capture the metabolic diversity of the obese population [i.e., ‘obese-metabolically unhealthy’ vs. ‘obese-metabolically healthy’ etc. (13, 27)] as well as being unable to separate the role of lipid accumulation from hyperinsulinemias and altered immune status. Recently, we showed that feeding mice a diet high in starch induced obesity and lipid accumulation in liver and skeletal muscle to levels similar to mice fed a high-fat diet, but that the high-starch diet group retained glucose tolerance and insulin sensitivity when compared with mice fed a high-fat diet (28). This approach provides a powerful platform to identify the molecular mechanisms that result in the storage of excess lipids and its links to insulin sensitivity, analogous to the “Athlete’s Paradox,” where highly insulin-sensitive, endurance-trained athletes have skeletal muscle lipid levels similar to that observed in insulin-resistant obese and type 2 diabetes subjects (29). In this study, we aimed to deploy this model—using mice fed either a high-fat diet (HFD), a high-starch diet (HStD), or Chow control—to determine changes in liver lipid droplet morphology and proteome associated with glucose tolerance from changes linked to liver lipid content.

## METHODS

### Animals

All surgical and experimental procedures performed were approved by the Animal Ethics Committee (University of Sydney) and were in accordance with the National Health and Medical Research Council of Australia's guidelines on animal experimentation.

Eight-week-old male C57BL/6_Arc_ mice were obtained from the Australian Animal Resource Centre (Perth, Australia). Mice were communally housed and maintained at 22 ± 1°C on a 12:12-h light-dark cycle with ad libitum access to food and water with corn cob bedding.

Mice were assigned to one of three diets as previously described (28). Briefly, the three diets were a standard Chow diet (11% fat, 23% protein and 66% carbohydrate, by calories; Specialty Feeds, Perth Australia), a high-fat diet [HFD; 60% fat, 20% carbohydrate (predominantly corn starch), 20% protein, by calories; based on Research Diets Formula No. D12492], or a high-starch diet [HStD; 20% protein, 20% fat, 60% carbohydrate (predominantly corn starch)] that were prepared in-house, where the macronutrients of the diets contained identical amounts (in total grams) of commercially available vitamins (AIN vitamins) to that of the Chow diet. The energy density of the standard chow diet, the HFD, and the HStD was 13 kJ/g, 13.4 kJ/g, and 9.42 kJ/g, respectively. All cages were maintained on their assigned diets for 12 wk. Food intake was performed on mice during week 10 by the daily weighing of food hoppers and food spillage in communally housed cages, and it was averaged to account for multiple mice per cage. Energy intake was calculated by multiplying the grams of food consumed with the energy density of the diet.

### Measurement of Physiological Parameters

Body composition was determined using the EchoMRI-500 (EchoMRI LLC, Houston) according to the manufacturers’ instructions, excluding body water.

Glucose tolerance was determined in mice that were fasted for 6 h (food removed at 8 AM). After a basal sample, an oral bolus of 50 mg of glucose (200 µL of 25% glucose solution in water) was administered, and glucose levels were measured at 15, 30, 45, 60, and 90 min post glucose load from tail vein blood using a glucose monitor (Accu-check Performa II, Roche Diagnostics, Australia). Insulin levels during the oral glucose tolerance test (Basal, 15- and 30-min post load) were measured in samples of whole blood collected from the tail using a mouse ultra-sensitive ELISA kit (Crystal Chem, Elk Grove Village, IL).

### Analytical Methods

Plasma insulin was measured using the Ultra-Sensitive Mouse Insulin ELISA kit from Crystal Chem, (USA). Liver triacylglycerols (TGs) were extracted using the method of Folch (30) and quantified using an enzymatic colorimetric method (GPO-PAP reagent; Roche Diagnostics).

### Lipid Droplet Isolation

Lipid droplet-enriched fractions were generated as previously published (31). Briefly, fresh livers were diced and then homogenized in ice-cold hypotonic lysis medium [HLM; 20 mM Tris-Cl pH 7.4, 1 mM EDTA, 10 mM NaF supplemented with protease and phosphatase inhibitors (Astral Scientific)] at a ratio of 4 mL medium/gram of tissue. Homogenates were then transferred to a 50 mL tube and centrifuged for 10 min at 1,000 *g* at 4°C (Beckman Coulter). The resulting supernatant was then transferred to microfuge tubes for storage at −80°C as whole liver lysates or to fresh ultracentrifuge tubes for further processing.

A sucrose gradient was prepared by mixing 3 mL of liver homogenate with 1 mL of HLM containing 60% sucrose in a 13.2 mL ultracentrifuge tube (Beckman Coulter), followed by 5 mL and then 4 mL of HLM buffer containing 5% and 0% sucrose, respectively. Samples were centrifuged for 30 min at 28,000 *g* at 4°C (P55ST2-636, Hitachi), with the rotor allowed to coast to a stop. The buoyant fraction (enriched with lipid droplets) was transferred to a 15 mL tube (Corning). Tubes containing the buoyant fraction were filled with 10 volumes of ice-cold acetone to delipidate the samples. Samples were incubated overnight at −20°C and centrifuged the next day for 1 h at 4,300 *g* at 4°C to pellet the proteins. A nitrogen sample concentrator was used to evaporate the residual acetone and dehydrate the pellet. Samples were then stored at −80°C until use.

### Immunoblotting

Whole liver lysates and lipid droplet-enriched fraction proteins were loaded on 10% SDS-PAGE gels and transferred onto polyvinylidene fluoride membranes (Merck). Membranes were incubated at room temperature for 1 h with blocking buffer [TBS, pH 4.5, with 0.1% Tween 20 (TBST) and 3% nonfat milk or BSA]. Membranes were incubated overnight at 4°C with a specific primary antibody in TBST and 3% BSA. Antibodies used were as follows: lipid droplet—ATGL (1:1,000, 2138S, CST) and PLIN2 (1:1,000, ab108323, Abcam); mitochondria—Mitomix (1:500, ab110413, Abcam—Mitosciences) and cytochrome c (1:1,000, 11940S, CST); cytosol—GAPDH (1:1,000, 2118S/5174S, CST); Golgi—GM130 (1:1,000, 12480S, CST). Subsequently, membranes were incubated with secondary HRP-coupled antibodies (rabbit: 7074P2, mouse: 7076S. CST), washed again then incubated with enhanced chemiluminescence reagent (Merck) prior to visualization using the ChemiDoc System (Bio-Rad Laboratories, Hercules). Data were analyzed using the ImageLab 5.2 v. software (Bio-Rad Laboratories, Hercules).

### Proteomic Analysis of Lipid Droplet Proteins

Lipid droplet proteins were resuspended in 4% sodium deoxycholate and 100 mM Tris-HCl (pH 7.5), and protein concentrations were quantified using a CBQCA Quantification kit (C-6667, Invitrogen) according to the manufacturer’s instructions. Samples were reduced using 10 mM TCEP and alkylated with 40 mM chloroacetamide at 95°C for 10 min. Following this, samples were diluted to 1% sodium deoxycholate using Tris-HCl (pH 8) and digested overnight with MS-grade trypsin (in 50 mM acetic acid) at 37°C while constantly shaking. Peptides were submitted to sample clean-up as described previously (32), with the only change being that only the aqueous phase was put through the tips. Samples were dried for 1 h at 45°C in a centrifugal evaporator and stored in 5% (vol/vol) formic acid at 4°C before LC/MS-MS analysis.

These peptides were analyzed using a Thermo Fisher Dionex RSLCnano UHPLC and directly added onto a 45 cm × 75 µm C-18 (Dr Maisch, Ammerbuch, Germany, 1.9 µm) fused silica analytical column with a 10 µm pulled tip, coupled to an online nano-spray ESI source. Peptides were resolved over a gradient from 5% ACN to 40% ACN running for 60 min with a flow rate of 300 nL/min. Peptides were ionized by electrospray ionization at 2.3 kV. Tandem mass spectrometry (MS/MS) analysis was performed using a Q-Exactive Plus mass spectrometer (Thermo Fisher) with higher energy collisional dissociation fragmentation.

Data-dependent acquisition was used with the acquisition of MS/MS spectra for the top 10 most abundant ions at any one point during the gradient. Raw data were analyzed using the quantitative proteomics software MaxQuant (33) (http://www.maxquant.org, v. 1.5.7.0). Peptide and protein level identifications were both set to a false discovery rate of 1% using a target-decoy-based strategy and proteins were filtered such that they had to have more than two razor and unique peptides. The database supplied to the search engine for peptide identifications contained the human UniProt database, downloaded on September 30, 2018, containing 42,170 protein sequence entries and the MaxQuant contaminants database. Mass tolerance was set to 4.5 ppm for precursor ions and MS/MS mass tolerance was 20 ppm. Enzyme specificity was set to trypsin (cleavage C-terminal to Lys and Arg) with a maximum of two missed cleavages permitted. Deamidation of Asn and Gln, oxidation of Met, pyro-Glu (with peptide N-term Gln), and protein N-terminal acetylation were set as variable modifications. N-ethylmaleimide on Cys was searched for as a fixed modification. The Max label-free quantification (LFQ) algorithm was used for LFQ, integrated into the MaxQuant environment (33, 34).

### Bioinformatic Analysis

Processing and statistical analysis of MaxQuant LFQ output was performed using the R software environment (v. 3.4.3) as previously described (35). For quantification, we applied a threshold that required an identified protein to be detected in at least seven of the 10 mice in any of the three diets. Statistical outputs were corrected for multiple comparisons using the Benjamini-Hochberg method (Supplemental Table S1). Protein-protein interaction networks and functional enrichment were analyzed using the STRING database (36) and Kyoto Encyclopedia of Genes and Genomes (KEGG) pathways.

Significantly different lipid droplet-associated proteins were filtered to identify proteins of interest by creating biologically relevant scenarios. Specifically, we identified proteins whose abundance correlated with liver TG levels using the following criteria: HFD versus Chow *P* ≤ 0.05, HStD versus Chow *P* ≤ 0.05, HStD versus HFD *P* ≥ 0.05. Next, we looked for proteins whose abundance correlated with glucose tolerance using the following criteria: HFD versus Chow *P* ≤ 0.05, Hi-ST versus Chow *P* ≥ 0.05, HStD versus HFD *P* ≤ 0.05.

### Liver Lipid Droplet Morphology

A portion of the liver was embedded in OCT and then cut into 10 µm sections using a cryotome FSE cryostat. A minimum of two cross-sections from different depths of the tissue was mounted on glass slide. Sections were covered with Oil Red O for 5 min and thereafter carefully rinsed under running tap water for 30 min. Next, slides were cover slipped with glycerol: water solution (9:1) and allowed to dry for 30 min before sealing the edges with nail polish.

Sections were visualized with the Zeiss Axio Vert.A1microscope, and images captured with a Zeiss Axiocam 105 camera using the Zeiss software. Quantification of liver lipid droplet number and size was performed using ImageJ software (NIH, Bethesda, MA). For lipid droplet number, liver sections were divided into defined squares (0.145 mm^2^) and all lipid droplets within this area were counted for each mouse (*n* = 3/group). For lipid droplet size, the same criteria were applied, and the average size for the total number of lipid droplets counted was calculated (37).

### Statistical Analysis

Statistical analyses were performed with GraphPad Prism 9.2.0 (GraphPad Software, San Diego, CA) or as described in Bioinformatic Analysis. Differences among groups were assessed with appropriate statistical tests noted in figure legends. *P* ≤ 0.05 was considered significant. Data are reported as means ± SE of at least three independent determinations. All authors had access to the study data and had reviewed and approved the final manuscript.

Our interests were focused on identifying molecular links to phenotype, not the influence of diet per se. As such, we removed two HStD-fed mice from our analyses as they failed to respond to the diet, as determined by their body weight, adiposity, and liver triacylglycerol (TG) levels (two SD away from the mean of the remaining mice, data not shown), and thereby failed to display the expected phenotype of the group. In addition, one HFD-fed mouse was also removed from our analyses because of technical issues with the proteomics (Supplemental Fig. S1*A*).

## RESULTS

### High-Fat and High-Starch Diet Feeding Leads to Increased Adiposity and Liver Triacylglycerol Content but Differs in Glucose Tolerance

**Figure 1.**
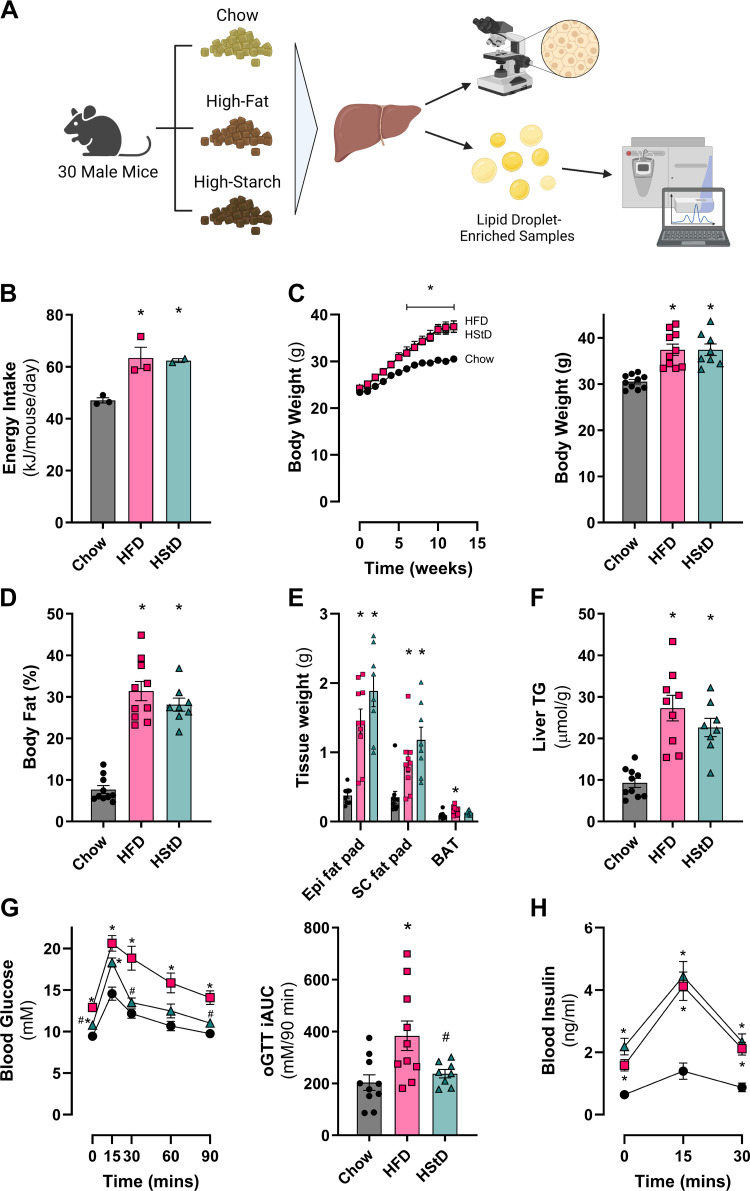
HStD and HFD similarly increases food intake, body weight, adiposity, and liver triacylglycerol levels but HStD retain glucose tolerance compared with HFD. *A*: experimental design of the morphometric and proteomic analyses of liver samples enriched for lipid droplets from mice fed Chow diet, high-fat diet (HFD), or high-starch diet (HStD). Created with BioRender.com. Average daily energy intake (*B*), body weight and end point body weight (*C*), body fat mass (*D*), tissue weights (*E*), liver triacylglycerols (TG) (*F*), blood glucose levels and incremental area under the curve (iAUC) for the oral glucose tolerance test (oGTT) after 12 wk of feeding (*G*), blood insulin levels for the oral glucose tolerance test after 12 wk of feeding (*H*). Data are presented as means ± SE; *n* = 3 cages for Chow and HFD (*B*), *n* = 2 cages for HStD; *n* = 10 mice for Chow, *n* = 9 mice for HFD, *n* = 8 mice for HStD (*C–H*). *P ≤ 0.05 vs. Chow; #*P* ≤ 0.05 vs. HFD by one-way ANOVA (*B* and *C right*, *D–F* and *G right*) or two-way ANOVA (*C left*, *G left*, *H*) followed by Tukey’s multiple comparisons test. ANOVA, analysis of variance.

In line with our previous observations (28), mice fed either an HFD or HStD had increased energy intake (Fig. 1*B*) and increased body weight (Fig. 1*C*) compared with mice fed Chow. Furthermore, mice fed either HFD or HStD had greater total fat mass (Fig. 1*D*) and epidydimal and subcutaneous fat pad mass (Fig. 1*E*), as well as liver TG content (Fig. 1*F*) compared with Chow-fed controls.

Despite the similarity in body weight, adiposity, and liver lipid content, mice fed HStD remained glucose tolerant, whereas mice fed HFD were glucose intolerant (Fig. 1*G*). The difference in glucose tolerance was not driven by differences in insulin levels (Fig. 1*H*; AUC, Chow: 49.4 ± 8.6 ng/mL/30 min; HFD: 136.4 ± 13.3 ng/mL/30 min; HStD: 160.9 ± 16.5 ng/mL/30 min). Collectively, these data demonstrate that HFD feeding led to increased adiposity, hepatic steatosis, and glucose intolerance, whereas HStD feeding resulted in similar levels of adiposity and hepatic steatosis but did not induce glucose intolerance. These observations are consistent with our previous report, where HStD feeding resulted in no evidence of hepatic insulin resistance, as determined using the gold-standard hyperinsulinemic-euglycemic clamp technique (28).

### Hepatic Lipid Droplet Morphology is Similar between Mice Fed HFD and HStD

**Figure 2.**
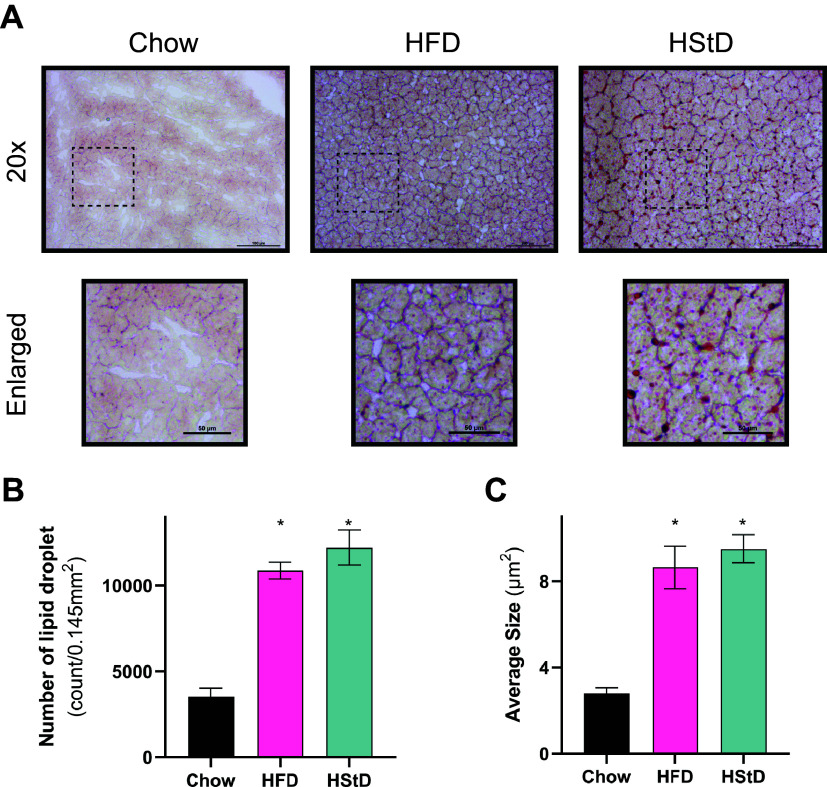
HStD and HFD similarly increase liver lipid droplet number and size compared with Chow. *A*: representative Oil Red-O stained mouse livers from mice fed Chow diet, high-fat diet (HFD), or high-starch diet (HStD) for 12 weeks. *B*: lipid droplet number per area and (*C*) size. Data are presented as means ± SE; up to four regions of interest quantified per mouse. *n* = 10 for Chow, *n* = 9 for HFD, *n* = 8 for HStD. *P ≤ 0.05 vs. Chow by one-way ANOVA followed by Tukey’s multiple comparisons test. Scale bars represent 100 µm (*top*) or 50 µm (*bottom*). ANOVA, analysis of variance.

The differences in whole body glucose metabolism between mice fed HFD and HStD, despite matched liver lipid levels, suggest that the excess lipid in cytosolic lipid droplets in the liver of mice fed HStD is benign with respect to insulin resistance, whereas mice fed HFD is not. As such, we determined whether differences in glucose tolerance were associated with liver lipid droplet morphology. Consistent with the biochemical measure of TG (Fig. 1*F*), an increased number and size of hepatic lipid droplets were observed in mice fed an HFD or HStD compared with mice fed a Chow diet (Fig. 2). However, there was no difference in the number and size of liver lipid droplets between HFD-fed mice and those provided with an HStD (Fig. 2). This suggests that the differences in whole body glucose metabolism between mice fed an HFD and HStD were not associated with changes in the morphology of liver lipid droplets, despite both groups exhibiting liver steatosis.

### High-Fat Diet Induces a Distinct Liver Lipid Droplet Proteome Compared with a High-Starch Diet

**Figure 3.**
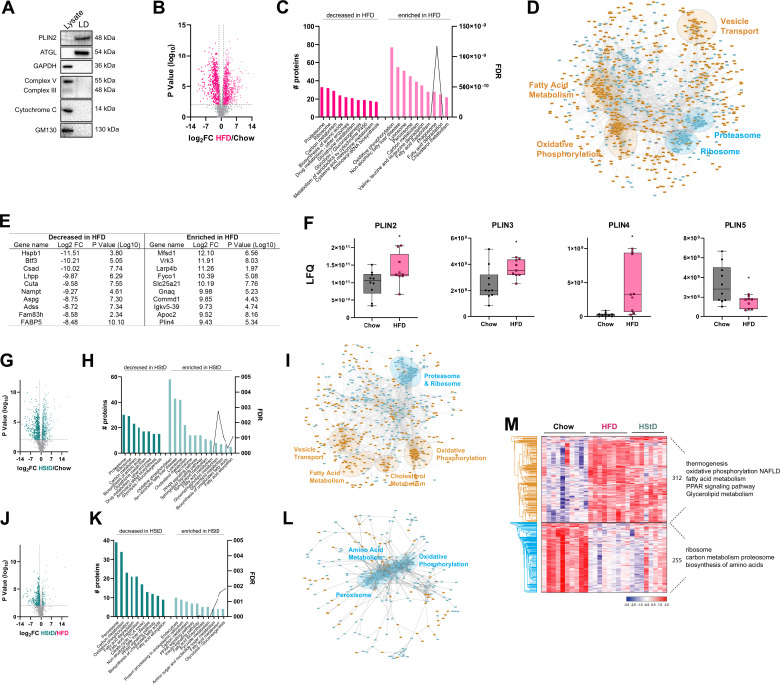
The proteome of liver lipid droplets is modified by HFD and HStD feeding. *A*: representative immunoblots of protein markers of organelles including lipid droplets (Plin2, ATGL), cytosol (GAPDH), mitochondria [protein subunits in the mitochondrial complexes (complex III-Core protein 2 and complex V-alpha subunit, cytochrome C)], and Golgi (GM130). Uncropped images Supplemental Fig. S3. *B*: volcano plot of lipid droplet-associated proteins in response to high-fat diet (HFD) feeding compared with Chow diet feeding. *C*: ontology analysis and (*D*) STRING analysis of significantly changed lipid droplet-associated proteins in response to HFD feeding compared with Chow diet feeding. *E*: list of proteins most significantly altered in abundance in the lipid droplet proteome following 12 wk of HFD feeding. *F*: box and whisker plots for specific proteins of interest. Each point represents protein abundance from an individual mouse. *G*: volcano plot of lipid droplet-associated proteins in response to high-starch diet (HStD) feeding compared with Chow diet feeding. *H*: ontology analysis and STRING analysis of significantly changed lipid droplet-associated proteins in response to HStD feeding compared with Chow diet feeding (*I*). *J*: volcano plot of lipid droplet-associated proteins in response to HStD feeding compared with HFD diet feeding. *K*: ontology analysis and STRING analysis of significantly changed lipid droplet-associated proteins in response to HStD feeding compared with HFD diet feeding (*L*). *M*: hierarchical clustering of label-free quantitation (LFQ) intensities of 567 significantly changed proteins (ANOVA, FDR 0.05) in the LD-enriched proteome revealed two clusters related to changes in response to HFD and HStD feeding. Numbers of proteins and selected enriched KEGG pathways (Fisher’s exact test, FDR 0.1) are indicated for marked clusters. *n* = 10 for Chow, *n* = 9 for HFD, *n* = 8 for HStD. Data in *F* are presented as box and whisker plots: median, interquartile range, and error bars representing Min to Max. *P ≤ 0.05 vs. Chow by *t* test. LFQ, label-free quantification. ANOVA, analysis of variance; FDR, false discovery rate.

We propose that the development of glucose intolerance is associated with molecular events that regulate the storage of excess lipids. Hence, we next quantified the proteomes of liver lipid droplets in mice fed our three diets. Lipid droplets were enriched by gently homogenizing fresh liver and sucrose gradient centrifugation (38). Enrichment was confirmed by immunoblot detection of established markers (Fig. 3*A*), and samples were delipidated prior to mass spectrometry analyses. We identified 1,968 proteins in LD-enriched samples from Chow-fed mice that met our inclusion criteria of a minimum of two peptides for each protein in at least 7 of 10 mice for Chow (Supplemental Table S2). Similarly, we identified 2,108 proteins in LD-enriched samples in seven of nine mice for HFD from HFD-fed mice and 2,030 proteins in seven of eight mice for HStD-fed mice (Supplemental Table S3). Of these identified proteins, 1,823 were common to all three groups (Supplemental Table S4; Supplemental Fig. S1*B*). The median Pearson correlation coefficient for the LD-associated proteome for each diet was 0.928 for Chow, 0.965 for HFD, and 0.927 for HStD (Supplemental Fig. S1*C*).

Next, a quantitative analysis of changes in LD protein abundance was performed using LFQ values. From this analysis, we identified 1,349 proteins with altered abundance in the LD-enriched samples from livers of HFD-fed animals compared with Chow, indicating that a significant proportion (74%) of the LD proteome is responsive to HFD feeding (Fig. 3*B*, Supplemental Table S5). Of these, 806 proteins in the LD-enriched fraction had increased abundance with HFD, whereas 543 had relatively lower abundance (Fig. 3*B*). KEGG pathway analysis of enriched proteins identified enrichment of components of NAFLD, fatty acid metabolism, and glycerolipid metabolism, while those proteins decreased in abundance were involved in carbon metabolism among other pathways (Fig. 3*C*). STRING analysis of protein-protein interaction (PPI) networks within the set of significantly changed LD-associated proteins (*n* = 817) in animals fed an HFD identified enrichment for components of fatty acid metabolism, vehicle transport, and oxidative phosphorylation and decreased proteasome and ribosome proteins (Fig. 3*D*).

Notably, several of the top 10 proteins that were most increased or decreased in liver LD-enriched fractions of HFD-fed mice included proteins that have been reported to influence liver lipid metabolism, including COMM domain-containing protein 1 (Commd1) (39), nicotinamide phosphoribosyltransferase (Nampt) (40), fatty acid binding protein 5 (FABP5) (40), and PLIN4 (41) (Fig. 3*E*). Furthermore, the protein levels of many known LD-associated regulators of TG lipolysis were, as expected, significantly altered in response to HFD feeding compared with Chow-fed mice, including PLIN2, PLIN3, PLIN4, PLIN5 (Fig. 3*F*), hormone-sensitive lipase (HSL; FC = 1.73, *P* = 0.016), G0S2 (FC = 1.58, *P* = 0.019), and ABHD5 (also known as CGI58; FC = −4.4, *P* < 0.0001), with the notable exception being ATGL (FC = 1.22, *P* = 0.19).

We next analyzed LD-enriched samples from mice fed the HStD to those from Chow-fed mice to identify changes in protein levels in another fatty liver model but one that retains insulin sensitivity. From this analysis, we identified 1,021 (56% proteins) of the proteome was differentially regulated in the LD-enriched fractions of the livers of mice an HStD compared with mice (Supplemental Table S6). Specifically, there were 531 proteins in the LD-enriched fraction that were increased, and 490 decreased in abundance in response to HStD feeding (Fig. 3*G*). Unsurprisingly for mice with fatty liver, the enriched LD-associated proteins were primarily involved in cholesterol, and fatty acid metabolism, glycerolipid metabolism, and fatty acid elongation and desaturation (Fig. 3*H*), which was distinct from HFD-fed mice (Fig. 3*C*). The increased abundance of proteins involved in fatty acid synthesis and modification is consistent with the notion that HStD-fed mice have to synthesize fatty acids, whereas HFD-fed mice have abundant access to fat from the diet (28). Those proteins decreased in abundance in HStD-fed mice were involved in carbon, glucose, and amino acid metabolism (Fig. 3*H*). PPI analysis of the significantly altered proteins identified clusters of proteins involved in fatty acid and cholesterol metabolism, oxidative phosphorylation, and decreased proteasome and ribosome proteins (Fig. 3*I*). Finally, we also identified 467 (26% proteins) significantly altered proteins in LD-enriched fractions of livers from HStD-fed mice compared with HFD mice (Supplemental Table S7), two groups with equal levels of liver TGs (Fig. 1*F*). Specifically, 116 proteins in the LD-enriched fraction were increased, and 351 decreased in abundance in response to HStD (Fig. 3*J*). The enriched LD-associated proteins in HStD-fed mice were primarily involved in fatty acid biosynthesis and metabolism, whereas those proteins that decreased in abundance were listed in the NAFLD and fatty acid metabolism KEGG pathways (Fig. 3*K*). We also observed pathways including carbon and fatty acid metabolism and PPAR signaling were increased and decreased, which can be explained by members of these pathways being differentially affected by HFD and HStD feeding. Significantly altered proteins underwent PPI analysis that identified clusters of proteins involved in amino acid metabolism, oxidative phosphorylation, and peroxisome that were downregulated in the HStD compared with HFD (Fig. 3*L*). Most interesting is the enrichment of proteins involved in oxidative phosphorylation in LD-enriched sampled from HFD-fed mice compared with HStD-fed mice, which suggests that there are dietary-specific effects on LD-mitochondrial interactions and, thereby, mitochondrial function (42, 43). Of note, there were striking differences in the patterns observed when comparing the STRING PPI analysis reported in Fig. 3, *D*, *I*, and *L*. These differences highlight a greater influence of lipid levels in mouse liver (i.e., Fig. 3, *D* and *I*) compared with glucose tolerance (Fig. 3*L*) on LD-associated proteome changes.

We next assessed the proteome data for all samples using ANOVA and identified 567 significantly changed proteins (Supplemental Table S8). Using hierarchical cluster analysis, we identified two clear clusters, one containing 312 proteins primarily involved in thermogenesis, oxidative phosphorylation, NAFLD, and fatty acid metabolism. In contrast, the second cluster had 255 proteins involved in the ribosome, carbon metabolism, proteasome, and biosynthesis of amino acids (Fig. 3*M*). It was not overly surprising that we only identified two clusters in our ANOVA analysis since the HStD induced a phenotype that exhibits similar traits to both Chow-fed animals and HFD-fed animals (Fig. 1). Collectively, we show that the proteome of liver lipid droplets of C57BL/6_Arc_ mice was altered in response to HFD and HStD feeding and that there were clear differences between these groups despite being equally obese and exhibiting similar levels of liver steatosis.

### The Fatty Liver Lipid Droplet Proteome

**Figure 4.**
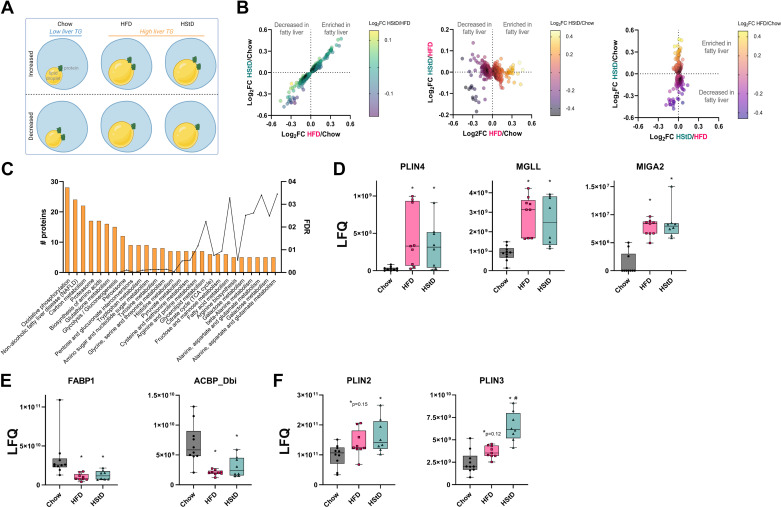
The fatty liver lipid droplet-associated proteome. *A*: biological scenario used to identify the fatty liver lipid droplet-associated proteome; increased/decreased in HFD compared with Chow (*P* ≤ 0.05) and increased/decreased in HStD compared with Chow (*P* ≤ 0.05) but not different between HStD and HFD (*P* ≥ 0.05). Created with BioRender.com *B*: the relationship of the Log_2_FC of proteins that were identified from the biological scenario data curation to identify fatty liver-associated changes in the lipid droplet proteome. *C*: ontology analysis of significantly changed lipid droplet-associated proteins that associate with fatty liver. Box-and-whisker plots for known lipid metabolism proteins that were enriched (*D*) and reduced (*E*) in the lipid droplet-associated proteins that associate with fatty liver. *F*: box-and-whisker plots for PLIN2 and PLIN3 abundance in the LD-enriched sampled from mice fed Chow, HFD, or HStD for 12 wk. Each point represents the protein abundance in lipid droplet-enriched fractions for an individual mouse. *n* = 10 for Chow, *n* = 9 for HFD, *n* = 8 for HStD. Data in (*D*)–(*F*) are presented as box and whisker plots: median, interquartile range, and error bars representing Min to Max. *P ≤ 0.05 vs. Chow; #*P* ≤ 0.05 vs. HFD by one-way ANOVA followed by Tukey’s Multiple Comparisons test. ANOVA, analysis of variance; HFD, high-fat diet; HStD, high-starch diet; LD, lipid droplet; LFQ, label-free quantification.

Our dietary approach provided a unique opportunity to uncouple the changes in the LD proteome of fatty liver from those changes associated with glucose tolerance and insulin sensitivity. First, we filtered our data through a biologically relevant scenario to identify proteins whose abundance correlated with liver TG levels (Fig. 4*A*); specifically, proteins that were increased/decreased in HFD compared with Chow (*P* ≤ 0.05) and increased/decreased in HStD compared with Chow (*P* ≤ 0.05) but not different between HStD and HFD (*P* ≥ 0.05). Using this approach, we identified 283 proteins that were increased and 285 proteins that were decreased in this scenario (Fig. 4*B*, Supplemental Table S9). KEGG pathway analysis of these proteins identified the enrichment of components of NAFLD and carbon, amino acid, and glycerolipid metabolism (Fig. 4*C*). Strikingly, many proteins known to be involved in liver lipid metabolism were also identified as being increased in the LD-enriched fractions of fatty liver, including PLIN4, monoacylglycerol lipase (MGLL), and mitoguardin 2 (MIGA2) (Fig. 4*D*), while fatty acid binding protein 1 (FABP1) and acyl-CoA-binding protein (ACBP_Dbi) were decreased (Fig. 4*E*). Other members of the PLIN family, PLIN2 and PLIN3, did not meet our criteria, but it is important to note that PLIN2 was increased in HStD samples compared with Chow but not significantly different between Chow and HFD (*P* = 0.15 by one-way ANOVA; Fig. 4*F*). PLIN3 was not significantly increased in HFD compared with Chow (*P* = 0.12 by one-way ANOVA) but increased in HStD compared with Chow and further enriched in LD-associated samples from HStD samples compared with HFD (by one-way ANOVA; Fig. 4*F*). Overall, we have defined the liver LD-associated proteome common to obese mice fed HFD and HStD, independent of the differences in glucose tolerance and insulin sensitivity between these groups.

### The Glucose-Intolerant Liver Lipid Droplet Proteome

**Figure 5.**
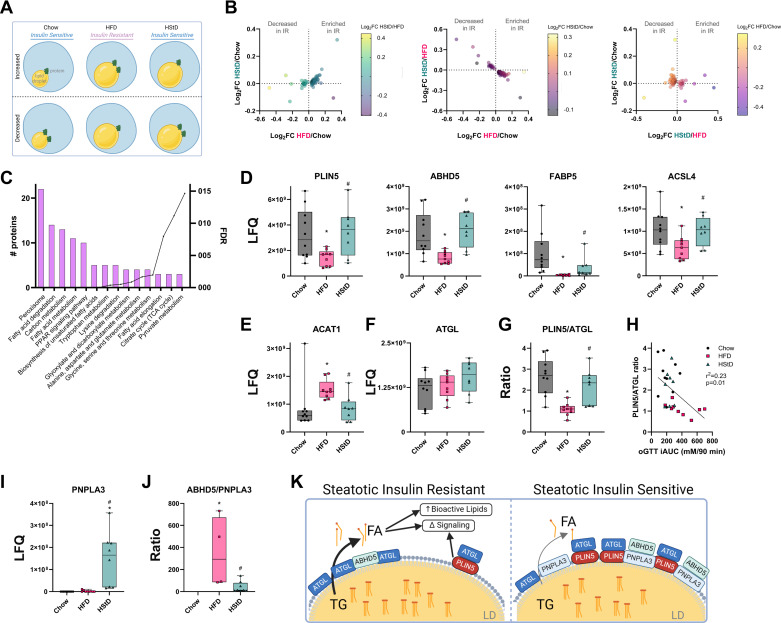
The liver lipid droplet-associated proteome that is associated with glucose tolerance and insulin sensitivity. *A*: biological scenario used to identify the liver lipid droplet-associated proteome that associates with insulin resistance; increased/decreased in HFD compared with Chow (*P* ≤ 0.05) and increased/decreased in HFD compared with HStD (*P* ≤ 0.05) but not different between HStD and Chow (*P* ≥ 0.05). Created with BioRender.com *B*: the relationship of the Log_2_FC of proteins that were identified from the biological scenario data curation to identify insulin resistance-associated changes in the lipid droplet proteome. *C*: ontology analysis of significantly changed liver lipid droplet-associated proteins that associate with glucose tolerance and insulin sensitivity. Box-and-whisker plots for known lipid metabolism proteins that were enriched (*D*) or decreased (*E*) in the lipid droplet-associated proteins that relate with glucose tolerance and insulin sensitivity. *F*: box-and-whisker plots for ATGL abundance and the PLIN5/ATGL ratio (*G*) of LFQ data in the LD-enriched sampled from mice fed Chow, HFD, or HStD for 12 weeks. *H*: linear regression analysis of PLIN5/ATGL ratio and incremental area under the curve (iAUC) for the oral glucose tolerance test (oGTT). *I*: box-and-whisker plots for APNPLA3 abundance and the ABHD5/PNPLA3 ratio (*J*) of LFQ data in the LD-enriched sampled from mice fed Chow, HFD, or HStD for 12 wk. Each point represents the protein abundance in lipid droplet-enriched fractions for an individual mouse. *K*: proposed model of changes in the liver lipid droplet-associated protein that aligns with glucose tolerance and insulin sensitivity. *n* = 10 for Chow, *n* = 9 for HFD, *n* = 8 for HStD. Data in *D*–*G* and *I*–*J* are presented as box and whisker plots: median, interquartile range and error bars representing Min to Max. *P ≤ 0.05 vs. Chow; #*P* ≤ 0.05 vs. HFD by one-way ANOVA followed by Tukey’s Multiple Comparisons test. ANOVA, analysis of variance; HFD, high-fat diet; HStD, high-starch diet; LD, lipid droplet; LFQ, label-free quantification.

Similar to our approach to identifying changes in protein levels of the LD-enriched fraction of liver from mice reported above, we filtered our data through another biologically relevant scenario to identify LD-associated proteins whose abundance correlated with glucose tolerance and insulin sensitivity (Fig. 5*A*); specifically, proteins that were increased/decreased in HFD compared with Chow (*P* ≤ 0.05) and increased/decreased in HFD compared with HStD (*P* ≤ 0.05) but not different between HStD and Chow (*P* ≥ 0.05). Using this approach, we identified 61 proteins that were increased and only 19 proteins that were decreased in this scenario (Fig. 5*B*; Supplemental Table S10). KEGG pathway analysis of these proteins identified the enrichment of components of fatty acid metabolism, degradation, elongation, and desaturation, as well as PPAR signaling (Fig. 5*C*). Many key proteins involved in liver lipid droplet homeostasis were identified as being decreased in the LD-enriched fractions of the liver of insulin-resistant mice, including PLIN5, ABHD5, fatty acid binding protein 4 (FABP4), and acyl-CoA synthetase long-chain family member 4 (ACSL4) (Fig. 5*D*), while acetyl-CoA acetyltransferase 1 (ACAT1), which catalyzes the esterification of cholesterol, was increased (Fig. 5*E*).

Of most interest were the changes in PLIN5, which acts as a gatekeeper for access to lipid droplet-contained substrates (44). So, we examined other known lipid metabolism proteins to put the changes in PLIN5 into context. From this, we observed no changes in the protein levels of DGAT2, G0S2 (Supplemental Fig. S2), and ATGL (Fig. 5*F*) between all three groups and a broad range of differences between groups for other lipid enzymes (Supplemental Fig. S2). ATGL activity is influenced by protein-protein interactions with its coactivators and co-suppressor (45–47), phosphorylation (48, 49) and translocation (50, 51), and access to its LD-contained substrates (44). Since we observed no difference in ATGL protein levels between groups (Fig. 5*F*), we proposed that changes in coregulator abundance at the lipid droplet may result in dysregulated ATGL activity in the insulin-resistant setting. Our first focus was on change in the ratio of PLIN5 and ATGL, which was reduced in LD-enriched samples from HFD-fed mice compared with both Chow and HStD groups (Fig. 5*G*). In fact, there was a negative relationship between the PLIN5/ATGL ratio and whole body glucose tolerance (Fig. 5*H*). Since PLIN5 blocks ATGL-mediated lipolysis by competitively binding to ABHD5 and disrupting the interaction between ABHD5 and ATGL (52), the change in the PLIN5/ATGL ratio in the glucose intolerant and insulin-resistant group (i.e., HFD) compared with the glucose tolerant and insulin sensitive groups (i.e., Chow and HStD) may lead to ATGL having increased access to its lipid droplet-contained substrates and altered regulation of lipolysis (52). In addition, the change in PLIN5 alone may lead to altered downstream signaling, such as SIRT1 and PPARα (53, 54).

Our second notable observation after examination of known lipid droplet and TG-related enzymes was the striking increase in PNPLA3 protein levels in lipid droplet-enriched samples from HStD-fed mice, compared with Chow and HFD-fed mice (Fig. 5*I*). Specifically, PNPLA3 was not detected in lipid droplet-enriched samples from the 10 Chow-fed mice and in only four out of nine HFD-fed mice. In contrast, it was detected in all eight HStD-fed mice. Similar to PLIN5, PNPLA3 competitively binds to ABHD5 to regulate ATGL activity (55, 56). So, we calculated the ABHD5/PNPLA3 ratio for samples with detectable PNPLA3 and determined that it was lower in HStD-fed mice than HFD-fed mice (Fig. 5*J*). This suggests that PNPLA3 is upregulated in HStD-fed mice in the face of lipid droplet expansion, likely leading to optimal ATGL activity homeostasis.

Combined, we propose that fatty liver-associated whole body glucose intolerance and insulin resistance are connected to dysregulated liver lipolysis due to changes in PLIN5 abundance and insufficient PNPLA3, likely leading to poorly controlled fatty acid metabolic fluxes that spill over to bioactive lipid synthesis that influence hepatic insulin action (57, 58), and downstream signaling (53, 54). This pattern is not observed in insulin-sensitive mice with fatty liver (Fig. 5*K*). Furthermore, the upregulation of PNPLA3 in HStD-fed mice in response to lipid droplet expansion due to high starch intake, likely prevents ABHD5 from stimulating ATGL activity and maintaining lipolytic homeostasis and thereby insulin action (Fig. 5*K*). This working model can form the basis for future investigations.

## DISCUSSION

The liver plays a vital role in nutrient homeostasis, and lipid accumulation in the liver is linked to the development of many pathologies, including NAFLD/NASH, hepatocellular carcinoma (HCC), insulin resistance, and type 2 diabetes. Studies exploring the mechanisms linking fatty liver to metabolic dysregulation, including insulin resistance, have predominantly used HFDs compared with Chow or defined controlled diets. Using a novel dietary approach that induced three metabolic phenotypes—*1*) lean, glucose tolerant, *2*) obese, steatotic liver and glucose intolerant, and *3*) obese, steatotic liver and glucose tolerant—we provide insights into the molecular events in lipid droplets of mice livers associated with lipid accumulation and those that associate with glucose intolerance and insulin resistance. From these observations, we propose that glucose intolerance and insulin resistance are associated with an imbalance in the lipolysome, consisting of lipolytic machinery (59), specifically the ratio of ATGL and PLIN5 and ABHD5 and PNPLA3, which are sustained or optimized in insulin-sensitive mice with fatty liver. Overall, we provide evidence for the possible mechanisms that occur at the lipid droplets accompanying the storage of excessive lipids in the livers of mice that are differentially associated with insulin sensitivity or resistance.

The relationships between fatty liver disease, perturbed metabolic physiology, and the development of pathologies such as type 2 diabetes, liver fibrosis, and HCC are complicated. Like so many aspects of biology, these relationships are not binary, in that, not all patients with fatty liver progress to NASH, type 2 diabetes, or HCC despite fatty liver being reported as a requisite for these conditions (5, 60, 61). Epidemiological data clearly show strong relationships between obesity and NAFLD—and the progression to NASH—regardless of metabolic health status (16, 62), while others have reported that metabolically healthy patients with obesity have less liver fibrosis compared with metabolically unhealthy patients with obesity (63). Likewise, a recent meta-analysis reported that patients with obesity who are metabolically healthy without fatty liver had an increased risk of developing type 2 diabetes [pooled relative risk 1.42 (95% CI 1.11–1.77)], but that this was significantly less than that of patients with obesity who are metabolically healthy with fatty liver [pooled relative risk 3.28 (95% CI 2.30–4.67)], compared with metabolically healthy nonoverweight subjects (64). Studies have also reported that patients with obesity who are insulin sensitive have a low degree of liver steatosis compared with those who are obese and insulin resistant (27, 65), which is a pattern that persists in follow-up assessment (66). The mechanisms that lead to fatty liver are many and include increased circulating fatty acid levels due to increased adiposity, increased de novo fatty acid synthesis, and insufficient increase in mitochondrial fatty acid oxidation, as well as gene variants [see review Bence and Birnbaum (67)]. Likewise, many mechanisms have been proposed to link fatty liver and metabolic dysfunction, including lipotoxic accumulation (including diacylglycerols and ceramides), oxidative stress, endoplasmic reticulum stress, impaired insulin signaling, and extrahepatic factors [see reviews (67–69)]. In general, these proposed mechanisms arise from studies involving humans with or without fatty liver, or from rodent studies using modified diets such as HFD and high sucrose/fructose.

Our HStD provides a novel model to explore the relationships between obesity, adiposity, fatty liver, and insulin resistance. Our detailed characterization of this model, compared with Chow and HFD-fed mice, included assessment of adiposity, glucose tolerance, insulin sensitivity by hyperinsulinemic-euglycemic clamp, and tissue lipids by mass spectrometry (28). In this study, we show that there was no relationship between lipid droplet morphology in the livers of HFD and HStD-fed mice and glucose intolerance. As such, mice fed HStD can store excess lipids in a manner that does not lead to metabolic dysregulation. This is analogous to the “Athlete’s Paradox,” where highly insulin-sensitive, endurance-trained athletes have skeletal muscle lipid levels similar to that observed in insulin-resistant obese and type 2 diabetes subjects (29). Therefore, this approach provides a unique and powerful model to explore the relationships between obesity, fatty liver, and insulin action.

The accumulation of lipid droplets in hepatocytes is a hallmark feature of fatty liver. Lipid droplets serve as temporary storage sites for excess lipids, including fatty acids (stored as TGs), ceramides (stored as acyl-ceramides), and retinols and sterols (as esters) (70). As such, the levels of lipids stored in lipid droplets, and thereby the size and number of lipid droplets, are the net effect of lipid synthesis and breakdown mechanisms that occur at the lipid droplet and the endoplasmic reticulum. Many studies have identified the lipid droplet proteome in rodent liver and in models of obesity and NAFLD (21–26). However, these studies predominantly compare two groups, for example, between Chow (control, low-fat diet) and HFD-fed animals or fasting and fasting-refed states, and so identify changes in the lipid droplet proteomes that are associated with multiple differences between two groups, such as adiposity, tissue and circulating lipid levels, circulating hormone levels, and immune status and including the HStD group that exhibits liver steatosis and glucose tolerance in our study design allowed us to identify changes in lipid droplet protein levels associated with fatty liver and those associated with glucose intolerance and insulin resistance. We identified 568 proteins (283 increased, 285 decreased) that were altered in fatty liver, but only 80 proteins (61 increased, 19 decreased) were associated with impaired glucose tolerance. It was unsurprising that fatty liver was associated with greater change in the lipid droplet proteome compared with glucose intolerance and insulin resistance. Many of the proteins that were enriched in the fatty liver proteome were involved in fatty acid metabolism and lipid droplet biology, including PLIN4 and MGLL, whereas FABP1 and ACBP_Dbi were less abundant. Consistent with Krahmer and colleagues (24), we observed changes in the levels of proteins involved in oxidative phosphorylation with HFD feeding, HStD feeding, and our fatty liver cluster. This suggests that increased lipid droplet number and size are associated with changes in interorganelle contacts with mitochondria (43).

Loss and gain of function studies of key LD homeostasis regulators, such as DGATs (71, 72), ATGL (73, 74), ABHD5 (75), HILPDA/HIG2 (76), PNPLA3 (77), and PLIN5 (78), have provided significant insights into links between LD biology and liver and whole body insulin action. However, these studies collectively demonstrate that there are complex relationships at play. In fact, both overexpression and knockdown of ATGL in mouse liver improved insulin action in a setting of HFD-induced insulin resistance (73, 74). Combined with the multidimensional changes that occur with HFD feeding and associating changes in the LD proteome with insulin resistance, it has been challenging to uncouple steatosis-dependent changes in protein levels at liver lipid droplets from those associated with insulin resistance. In this study, the novel inclusion of the HStD group enabled the identification of 80 proteins whose abundance was associated with insulin resistance and glucose intolerance. Notable proteins included many that are involved in fatty acid metabolism and lipid droplet homeostasis, such as ABHD5, FABP5, ASCL4, and PLIN5. In the context of unchanged ATGL levels, the altered abundance of PLIN5 in LD is fascinating, as PLIN5 blocks ATGL-mediated lipolysis by competitively binding to ABHD5 and disrupting the interaction between ABHD5 and ATGL (52). We observed a reduction in the ATGL to PLIN5 ratio in the insulin-resistant steatotic HFD group but not in the insulin-sensitive steatotic HStD and insulin-sensitive Chow groups, indicative of less PLIN5 to prevent ATGL TG hydrolase activity. As such, we predict that in the insulin-resistant setting, dysregulated lipolysis likely contributes intracellular fatty acids to support bioactive lipid synthesis, such as ceramides, and impaired insulin action (28). This predicted increase in lipolytic activity may lead to reduced TG levels, yet it is conceivable that the synthesis and breakdown rates are matched to sustain TG levels. However, the TG synthesis and breakdown flux differ between the HFD and HStD groups. PLIN5 plays important roles in other aspects of fatty acid metabolism, which are regulated by the phosphorylation of Ser155 as well as being tissue/cell-specific (79), and can influence a range of downstream signaling pathways (53, 54). In vivo quantification of hepatic lipolytic activity and PLIN5 function to validate this hypothesis is required but remains technically challenging to perform. Nonetheless, we provide novel insights into the proteomic changes at the LDs of livers of mice that are associated with insulin resistance and separate from changes due to increased TG levels.

PNPLA3 is a member of a family of proteins with lipase activity, including ATGL (encoded by *PNPLA2*), yet its precise function in neutral lipids metabolism is controversial and complex (80). To date, the majority of research interest in PNPLA3 has centered on understanding the mechanisms that link the rs738409[G] (encoding I148M) polymorphism and NAFLD progression and has resulted in an appreciation that loss of PNPLA3-mediated TG hydrolase activity alone is unlikely to cause hepatic steatosis, as ablation of wild-type PNPLA3 does not alter TG levels (77). Our studies identified a striking increase in PNPLA3 protein levels only in lipid droplet-enriched samples from mice fed a HStD. This aligns with reports that PNPLA3 expression is increased in mice fed high-sucrose (81) and high-carbohydrate diets (82, 83). Similar to PLIN5-regulation of TG hydrolysis, PNPLA3 coprecipitates with endogenous ABHD5 (55), competes with ATGL for ABHD5, and overexpression of PNPLA3 suppresses ABHD5-dependent lipolysis in brown adipocytes (56). In fact, it has been reported that the interaction between ABHD5 and PNPLA3 is much stronger than its interaction with ATGL, and is similar to the interaction of ABHD5 with PLIN1 or PLIN5, which are well-established suppressors of the interaction of ABHD5 with ATGL in adipocytes (84, 85). Our results suggest that the upregulation of PNPLA3 on liver lipid droplets in response to high starch consumption may serve as a protective mechanism to maintain optimal regulation of TG hydrolysis and ATGL function. This likely prevents the development of insulin resistance and glucose intolerance and broader metabolic dysfunction, and likely contributes to the benign storage of excessive lipids in the livers of mice.

Our findings demonstrate distinct changes in the liver LD proteome associated with the development of fatty liver that differ from those associated with insulin resistance. Furthermore, these changes occurred in settings with no LD number or size changes. Combined with our comprehensive metabolic characterization of the HStD and HFD models (28), these data provide new insights into the complex relationships between ectopic lipid accumulation, LD biology, and insulin resistance.

## DATA AVAILABILITY

The datasets generated during the current study are available from the corresponding author upon reasonable request.

## SUPPLEMENTAL DATA

10.6084/m9.figshare.25467769 Supplemental Tables S1–10: https://doi.org/10.25833/ykzp-ps30 and Supplemental Figs. S1–S3: https://doi.org/10.6084/m9.figshare.25467769.

## GRANTS

A.J.H. is supported by a Robinson Fellowship and funding from the University of Sydney.

## DISCLOSURES

No conflicts of interest, financial or otherwise, are declared by the authors.

## AUTHOR CONTRIBUTIONS

G.J.C., M.L., D.N.S., A.E.B., and A.J.H. conceived and designed research; A.V.W., D.J.H., S.R.N., M.F.H.-S., J.L., M.H., T.P., G.J.C., and A.J.H. performed experiments; A.V.W., D.J.H., S.R.N., M.F.H.-S., J.L., M.H., T.P., G.J.C., D.N.S., A.E.B., and A.J.H. analyzed data; A.V.W., D.J.H., S.R.N., M.F.H.-S., G.J.C., M.L. D.N.S., A.E.B., and A.J.H. interpreted results of experiments; A.V.W., D.J.H., D.N.S., A.E.B., and A.J.H. prepared figures; D.N.S., A.E.B., and A.J.H. drafted manuscript; A.V.W., D.J.H., S.R.N., M.F.H.-S., J.L., M.H., T.P., G.J.C., M.L., D.N.S., A.E.B., and A.J.H. edited and revised manuscript; A.V.W., D.J.H., S.R.N., M.F.H.-S., J.L., M.H., T.P., G.J.C., M.L., D.N.S., A.E.B., and A.J.H. approved final version of manuscript.
